# Characterisation of four novel bacteriophages targeting carbapenem-resistant *Klebsiella pneumoniae* and their lytic activity alone and in combination

**DOI:** 10.1016/j.crmicr.2025.100509

**Published:** 2025-11-10

**Authors:** Elisa Fausti, Andrea Bonacorsi, Novella Cesta, Cesira Giordano, Simona Barnini, Magda Marchetti, Claudia Campobasso, Rob Lavigne, Anna Altieri, Cartesio D’Agostini, Marco Iannetta, Vincenzo Malagnino, Arianna Tavanti, Loredana Sarmati, Mariagrazia Di Luca

**Affiliations:** a Department of Biology, University of Pisa, Via San Zeno 37, Pisa 56127, Italy; b Microbiology, Immunology, Infectious Diseases, and Transplants (MIMIT), University of Rome Tor Vergata, Rome, Italy; c Infectious Diseases Unit, Azienda Ospedaliera Universitaria Pisana, Pisa, Italy; d Microbiology Unit, Azienda Ospedaliera Universitaria Pisana, Pisa, Italy; e National Centre for Innovative Technologies in Public Health, Istituto Superiore di Sanità, Rome, Italy; f Department of Biosystems, Laboratory of Gene Technology, KU Leuven, Belgium; g Laboratory of Clinical Microbiology, Policlinico Tor Vergata, Rome, Italy; h Clinical Infectious Diseases, Department of System Medicine, Tor Vergata University, Rome, Italy

**Keywords:** Bacteriophage, Phage therapy, *Klebsiella pneumoniae*, Antibiotic resistance, Carbapenem resistance, Phage cocktail, Antibiofilm activity

## Abstract

*Klebsiella pneumoniae* is a nosocomial pathogen with rising levels of antibiotic resistance, increasing interest in bacteriophage therapy as an adjunct or alternative treatment. This study aimed to isolate and characterise bacteriophages active against multidrug-resistant *K. pneumoniae* and evaluate their efficacy. Four lytic phages, Kilian, Trimon, Jurek, and Olmo were isolated from water sources using *K. pneumoniae* ATCC BAA-2146 as host. Genome analysis revealed no known lysogeny, antibiotic resistance, or virulence-associated genes, supporting their suitability for therapeutic use. Electron microscopy classified Kilian, Trimon, and Jurek as siphoviruses, and Olmo as a myovirus. All phages showed rapid adsorption (2.5–5 min), short latent periods (5–15 min), and variable burst sizes (22–474 PFU/cell). The phages exhibited a narrow host range, collectively infecting 13 out of 90 tested *K. pneumoniae* clinical isolates, including carbapenemase producers and high-risk clones (ST11, ST512, ST147, ST307, ST37). The phages were then formulated into a cocktail and tested *in vitro*, showing enhanced and sustained bacterial growth suppression compared to single phages, with strain- and multiplicity of infection-dependent effects. Antibiofilm activity was assessed on preformed biofilms grown on porous glass beads. Three hours after treatment, both individual phages and the cocktail reduced viable biofilm-associated cells >4-log_10_ compared to untreated controls. Finally, high titers of purified phages obtained by cesium chloride gradient ultracentrifugation showed no adverse effects on *Galleria mellonella* viability, indicating a safe profile. These findings support the potential of phage combinations to target multidrug-resistant and biofilm-associated *K. pneumoniae* infections.

## Highlights

• Four newly isolated lytic phages showed no antibiotic resistance or virulence genes.
• Phages demonstrated rapid adsorption, short latent period and variable burst size.
• Phages were active against carbapenemase-producing *Klebsiella pneumoniae* strains.
• Phage cocktail enhanced lytic activity compared to individual phages.
• The antibiofilm activity suggests potential use in biofilm-related infection.

## Graphical abstract

**Figure:**
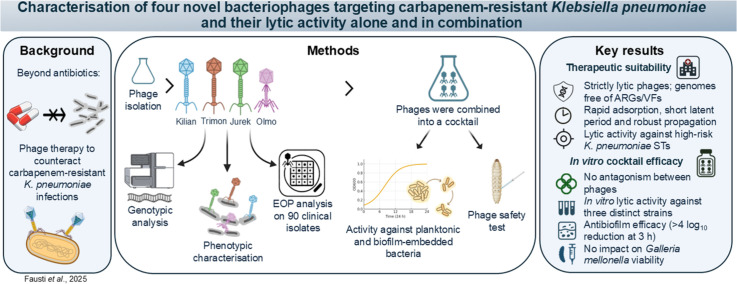

## 1 Introduction

*Klebsiella pneumoniae* is an opportunistic pathogen responsible for severe infections such as pneumonia, bloodstream and urinary tract infections (Paczosa and Mecsas, 2016). Its ability to form biofilms significantly enhances virulence and complicates treatment by providing a protective barrier against both the host immune system and antimicrobial agents. Most clinically relevant *K. pneumoniae* biofilms develop on catheters and the surface of implanted medical devices (Guerra et al., 2022). In addition, siderophores, fimbriae, efflux pumps, and hypermucoviscosity all contribute to persistence and immune evasion, further increasing its clinical impact (Wang et al., 2020).

A major challenge in treating *K. pneumoniae*-associated infections lies in its genetic capacity to develop antibiotic resistance. This pathogen has acquired resistance to many first-line antibiotics, including beta-lactams and aminoglycosides, limiting effective treatment options (Martin and Bachman, 2018). Of particular concern are carbapenemase-producing *K. pneumoniae* strains, which exhibit resistance to carbapenems, a class of antibiotics often considered a last resort for patient treatment. Key carbapenemase enzymes include VIM (Verona integron-encoded metallo-β-lactamase), KPC (*K. pneumoniae* carbapenemase), and NDM (New Delhi metallo-β-lactamase). These strains are frequently associated with high-risk sequence types (STs), such as ST258, ST11, and ST147, which are circulating globally and associated with nosocomial outbreaks, posing significant public health challenges. In Italy, sequence type ST147, associated with strains producing New Delhi metallo-β-lactamase (NDM), has been implicated in recent outbreaks. In Italy, sequence type ST147 of *Klebsiella pneumoniae*, producing New Delhi metallo-β-lactamase (NDM), has emerged as a major contributor to recent outbreaks. A large outbreak in Tuscany (November 2018–October 2019) involved 1645 NDM-positive clinical isolates, 90.9 % of which were *K. pneumoniae* (Tavoschi et al., 2020). Genomic analyses of ST147/NDM-1 K*. pneumoniae* revealed a complex resistome and virulome, driven by the acquisition of multiple mobile genetic elements, including an integrative conjugative element encoding yersiniabactin, an FIB(pQil)-type plasmid carrying *bla*NDM-1, and a chimeric plasmid with both resistance and virulence genes. The clone was resistant to nearly all antibiotics except cefiderocol, aztreonam-avibactam, colistin, and fosfomycin. Virulence varied among isolates, as shown in *Galleria mellonella* and serum killing assays, and resistance phenotypes were linked to mutations in chromosomal loci such as *csrD, pal*, and *ramR* (Di Pilato et al., 2022).

Bacteriophages, viruses that specifically infect bacteria, have emerged as a promising strategy to combat multidrug-resistant (MDR) pathogens like *K. pneumoniae* (Herridge et al., 2025; Kulshrestha et al., 2024)*.* Phages exhibit a unique mode of action by lysing bacterial cells in a highly specific manner (at the strain level), minimizing collateral damage to the microbiota and reducing the likelihood of systemic side effects. Furthermore, bacteriophages are self-amplifying, increasing in number at the infection site, and exert selective pressure on bacteria that can lead to evolutionary trade-off events, such as a reduced virulence or restored antibiotic sensitivity (Strathdee et al., 2023).

Phage therapy offers two key approaches. The first involves personalized phage preparations, typically composed of a phage cocktail specifically tailored to target the bacterial strain isolated from the patient. This specificity addresses the narrow host range typical of many phages and ensures efficacy against the clinical isolate of interest (Sawa et al., 2024). The second approach focuses on the development of broad-spectrum phage cocktails, which combine multiple phages to extend the host range. These cocktails are designed not only to target a variety of bacterial strains but also to delay the emergence of phage-resistant variants, making them suitable for treating multiple patients with similar infections (Yoo et al., 2024).

*In vitro* studies have established the efficacy of phage cocktails against *K. pneumoniae*, demonstrating their ability to broaden the host range and suppress the development of phage resistance (Yoo et al., 2024). Cocktails have also shown significant success in reducing bacterial viability in biofilm-associated infections (Chen et al., 2024a). Preclinical models further support these findings, highlighting the potential of phage cocktails to disrupt biofilms and improve therapeutic outcomes (Chen et al., 2024a). Beyond preclinical evidence, the outcome obtained in patient treatments are promising. Most of the scientific literature highlights the efficacy of personalized phage therapies, used in combination with antibiotic administration. For instance, a 57-year-old patient with persistent colonization of *K. pneumoniae* in the gastrointestinal tract, urine, and a permanent external invasive device received a three-week personalized bacteriophage therapy (phage vB_KpnM_GF) *via* oral and intra-rectal administration, which led to the complete eradication of the pathogen without recurrence (Corbellino et al., 2020)​. In another case report, a 62-year-old diabetic patient with a chronic prosthetic knee infection due to *K. pneumoniae* received intravenous phage therapy (phage KpJH46Φ2), leading to the resolution of infection and sustained clinical recovery at 34 weeks post-treatment​ (Cano et al., 2021). Moreover, a recent retrospective analysis of personalized phage therapy targeting various multidrug-resistant bacterial infections, including six cases of *K. pneumoniae*, reported clinical improvement in 77.2 % of cases and bacterial eradication in 61.3 % (Pirnay et al., 2024). Among the reported cases, a 30-year-old bombing victim with a fracture-related *K. pneumoniae* infection -resistant to all available antibiotics was successfully treated with a pre-adapted bacteriophage (phage vB_KpnM_M1) in combination with meropenem, colistin, and later ceftazidime/avibactam, resulting in significant clinical and microbiological improvement (Eskenazi et al., 2022)​. In literature, the only documented case of bacteriophage therapy used as a standalone treatment, without concomitant antibiotics, describes a 70-year-old female kidney transplant recipient with a recurrent urinary tract infection caused by *K. pneumoniae* producing extended-spectrum beta-lactamase (ESBL). Specifically, the patient underwent a four-week intravenous phage therapy regimen using a cocktail of three lytic phages (Metamorpho, Mineola, and pKp20). The treatment successfully eradicated the ESBL strain, with no recurrence for 206 days post-therapy. Subsequent *K. pneumoniae* infections were antibiotic-sensitive and manageable with oral therapy, eliminating the need for intravenous antibiotics (Le et al., 2023).

This evidence underscores the potential of both individual phages and phage cocktails as effective tools in tackling biofilm-associated and systemic infections caused by MDR *K. pneumoniae*. The discovery of novel bacteriophages and their strategic combination into customized cocktails is essential for both standardized and personalized approaches to phage therapy. These approaches leverage the high specificity of phages to selectively target resistant bacterial strains while minimizing off-target effects. This strategy not only enhances treatment efficacy but also contributes to addressing the escalating threat of antimicrobial resistance.

In this context, the aim of this study was to perform the genotypic and phenotypic characterisation of four *K. pneumoniae* bacteriophages isolated from water samples in Tuscany, Italy. Additionally, the antibacterial efficacy of the phages, administered either individually or as a cocktail, was evaluated against both planktonic and biofilm-associated cells of MDR *K. pneumoniae* strains. Finally, the safety profile of the phages was assessed using *Galleria mellonella* larvae as an *in vivo* model.

## 2 Materials and methods

### 2.1 K. pneumoniae strains

In this study, K. pneumoniae ATCC BAA-2146 (ST11) (Hudson et al., 2014) was used as host strain for both phage isolation and characterisation. A collection of 89 clinical strains of K. pneumoniae isolated in different Italian Hospitals were used. They include strains producing carbapenemase (KPC), New Delhi metallo-beta-lactamase (NDM), and Verona Integron-encoded metallo-beta-lactamase (VIM), with sequence types (STs) 37, 147, 307, and 512. Among them, 34 were NDM-producing strains: 33 belonging to ST147 and isolated from Cisanello Hospital (Azienda Ospedaliera Universitaria Pisana, Pisa, Italy) and one strain was collected Policlinic Tor Vergata Hospital (Rome, Italy). KPC-producing isolates represented the largest group, with 46 strains. Specifically, four KPC-producing isolates with ST512, two with ST307, and three with ST37 were collected from Cisanello Hospital, four ST307 isolates were obtained from Policlinic San Matteo Hospital (Pavia, Italy), 31 isolates were collected from Policlinic Tor Vergata Hospital, one from Borsellino Hospital (Marsala, Italy) and another from Santa Maria della Misericordia Hospital (Udine, Italy). Seven VIM-producing isolates were collected from Policlinic Tor Vergata Hospital. Finally, two isolates without genotype and sequence type information were included: one collected from Policlinic San Matteo Hospital and one from Policlinic Tor Vergata Hospital. A collection of 14 *Escherichia coli* strains from the Microbiology Laboratory, Department of Biology, University of Pisa (Italy), was used to verify the host range of the *K. pneumoniae* phages. All bacterial strains were cultured at 37 °C in Luria-Bertani (LB) broth and on LB agar plates (Sigma-Aldrich, St. Louis, Missouri, USA). Bacterial stocks were stored at −80 °C using cryovial bead preservation systems (SARSTEDT AG & Co, Germany).

### 2.2 Phage isolation

Phages were isolated with previously described protocols, with modifications (Townsend et al., 2021; Turchi et al., 2024). Either river or wastewater samples collected from Tuscany (Italy) were centrifuged at 5000 × *g* for 20 min at 4 °C and supernatants were filtered (0.22 µm; Primo Syringe Filters, Euroclone). Then, 10 mL supernatants were mixed with an equal volume of two-fold concentrated LB broth supplemented with 10 mM of CaCl_2_ and 10 mM of MgSO_4_ and incubated with 100 µL of an overnight (O/N) culture of *K. pneumoniae* ATCC BAA-2146 at 37 °C, while shaking (80 rpm) in an Edmund Buhler GmhH-KS15 incubator. After 24 h of incubation, enriched samples were centrifuged at 5000 × *g* for 20 min at 4 °C. The filtered supernatant was spotted onto a bacterial lawn of *K. pneumoniae* ATCC BAA-2146 strain prepared by pouring a mix of 50 µL of the O/N bacterial culture and 5 mL of 0.7 % LB soft agar on LB agar plates. Plates were incubated O/N at 37 °C. Enriched samples resulting in a lysis of the bacterial lawn were streaked again to isolate single plaques. Single plaques were then picked with a sterile tip and transferred to a bacterial lawn for phage propagation (Van Charante et al., 2019). Five mL of saline magnesium (SM) buffer (5.8 g/L NaCl, 2 g/L MgSO_4_, 50 mL/L of 1 M Tris–HCl, pH 7.5) were added to the plate to elute phages. The eluate was collected from the plates, centrifuged at 5000 × *g* for 20 min at 4 °C and filtered (0.22 µm). Phage titer was determined by double-layer agar spot assay of ten-fold serial dilutions of the eluted phages.

### 2.3 Phage genome extraction, sequencing and bioinformatic analysis

To remove bacterial DNA, 180 μL of phage lysate were mixed with 20 μL of 10 × DNaseI buffer and 10 μL of RNA-free DNaseI (1 U/μL) (Thermo Fisher Scientific) and incubated for 30 min at 37 °C. Twenty μL of 50 mM EDTA and 1 % SDS were added to inactivate the DNaseI. Ten μL of proteinase K (> 600 U/μL) (Thermo Fisher Scientific) were added and the samples were incubated for 45 min at 55 °C. Then, the DNA was purified using the Zymo Research DNA Clean&Concentrator™ kit. The eluted DNA was quantified using the NanoDrop™ Lite Spectrophotometer (Thermo Scientific™, Milan, Italy) and stored at −20 °C until use. For whole genome sequencing by Illumina, libraries were prepared using the Nextera Flex DNA Library Kit. For each sample, raw data were submitted to the BV-BRC online platform v3.6.12 (Wattam et al., 2017) and the assembly of phage genomes was performed using Unicycler v0.4.8 (Wick et al., 2017). BLASTn (Altschul et al., 1990) was used to screen the NCBI nucleotide collection database to find similar sequences to the phage genome sequence. Any new species were determined according to Turner and colleagues (Turner et al., 2021). The final FASTA sequences were provided to the BV-BRC platform to run genome annotations using RASTtk (Brettin et al., 2015), followed by manual functional annotation by comparing BV-BRC predicted coding sequences (CDSs) against the non-redundant GenBank protein database (Kelley and Sternberg, 2009) using BLASTp (Altschul et al., 2005).The potential presence of genes associated with virulence and antibiotic resistance in the phage genome was analyzed by ABRicate (Seemann, 2016) using the Comprehensive Antibiotic Resistance Database (CARD) (Jia et al., 2017), ResFinder (Bortolaia et al., 2020), NCBI (Sayers et al., 2021) and the Virulence Factor Database (VFDB) (Liu et al., 2019). The phylogenetic tree was generated using ViPTree by selecting phage genomes with the highest similarity, based on Similarity Genome (S_G_) between 0.70 and 1 (Nishimura et al., 2017). Intergenomic distances were assessed between each phage using VIRIDIC (Moraru et al., 2020). The packaging strategy of the viral progeny was investigated by comparing the terminase sequences of the newly isolated phages with those of phages with characterized genome packaging systems (Merrill et al., 2016).

### 2.4 Transmission electron microscopy (TEM)

A 10 μL aliquot of each sample was absorbed onto a formvar/carbon coated 400-mesh copper grid (Agar Scientific, Essex, UK). The excess of the sample was removed by filter paper and 10 μL of 2 % (w/v) phosphotungstic acid (pH 7.0) were added to the grid and incubated for 30 s. Negatively stained samples were examined with a FEI/Philips EM 208S transmission electron microscope (FEI, Eindhoven, Netherlands), operating at an accelerating voltage of 100 kV, equipped with acquisition system/Megaview SIS camera (Olympus, Hamburg, Germany). The images acquired were analysed using Fiji V1.0 (Schindelin et al., 2012).

### 2.5 Host range determination and efficiency of plating (EOP)

The phage host range was analysed by spot assay (Khan Mirzaei and Nilsson, 2015). Briefly, 10 µL of each phage lysate, at a starting concentration of 10¹⁰ PFU/mL, were ten-fold serially diluted and spotted onto freshly prepared bacterial lawns. Plates were incubated O/N at 37 °C. Plaques were counted, and the efficiency of plating (EOP) was calculated (Kutter, 2009). In some cases, although no countable plaques could be appreciated, lysis halos were detected at high phage concentrations. To assess the capacity of these phages to efficiently infect and propagate within the specific bacterial strains, exponential phase bacterial cells were coincubated with phages at a multiplicity of infection (MOI) of 0.1 at 37 °C for 24 h under shaking conditions (110 rpm). Phage titers were subsequently determined by spot assay.

### 2.6 Phage adsorption assay and one-step growth curve

Adsorption assays were optimized for *K. pneumoniae* according to previously described protocols (Hyman and Abedon, 2009; Kropinski, 2018), with some modification. *K. pneumoniae* ATCC BAA-2146 strain was cultured until exponential phase of growth, at approximately 1 × 10^8^ colony forming units (CFU)/mL, and the phage suspension was then added at a MOI of 0.001 (∼10^5^ PFU/mL). The mixture was incubated at 37 °C under shaking conditions (110 rpm). At each time point (0, 2.5, 5, 7.5, 10, 12.5, 15, 20, 25 and 30 min), aliquots of 200 μL were collected and immediately centrifuged at 12,000 × *g* for 2 min. Then, the supernatant containing unabsorbed phages was diluted and spotted using the soft agar overlay method. Adsorption data were represented as a decrease in unabsorbed phages overtime, expressed in percentage, as follow:

$$\frac{Titer\;of\;free\;phage\;per\;timepoint}{Initial\;titer\;of\;phage}x\;100\%$$

The one-step growth curve of the phages was determined following a previously described protocol (Gao et al., 2020), with minor modifications. Briefly, 5 mL of K. pneumoniae ATCC BAA-2146 in the exponential phase of growth (about 10^8^ CFU/mL) were mixed with phages (MOI of 0.001) and incubated at 37 °C for either 2.5 or 5 min, as determined for each phage by the adsorption curve. After incubation, the suspension was centrifuged at 5000 × *g* for five minutes at 4 °C. The supernatant was discarded, and the pellet was resuspended in 5 mL of fresh medium to remove any unabsorbed phage. Aliquots were taken at time zero, every five minutes for 20 min, and then every 10 min over a period of 130 min. Samples were immediately centrifuged at 12,000 × *g* for two minutes, serially diluted and spotted using the soft agar overlay method. The phage plaques were counted the following day, and the PFU/mL values were plotted over time. The burst size was calculated as follows:

$$Burst\;size=\frac{\frac{Phage\;titer\;after\;the\;burst}{Initial\;phage\;titer}}{\frac{CFUs}{mL}of\;infected\;cells}$$

### 2.7 Assessment of phage lytic activity by optical density measurements

The lytic activity of phages was evaluated by monitoring the optical density at 600 nm (OD_600_) overtime, every hour up to 24 h, using a microtiter plate reader (Sunrise Rainbow Thermos RC, TECAN, Austria). Two hundred fifty µL of an O/N K. pneumoniae culture were inoculated in 5 mL of fresh LB broth until an OD_600_ of approximately 0.4 was reached. Then, the bacterial culture was diluted to approximately 10^6^ CFU/mL and incubated with phages at different MOIs (0.1, 1, and 10) of phages either individually or as part of a four-phage cocktail (phage ratio 1:1:1:1). The OD_600_ of the LB broth, used as blank, was subtracted from OD_600_ values obtained from each sample and the lytic activity of phages was compared to the untreated growth control.

### 2.8 Phage activity on *K. pneumoniae* ATCC BAA-2146 biofilm

*K. pneumoniae* ATCC BAA-2146 biofilm was formed on porous glass beads (ROBU® Glasfilter-Geraete GmbH, Hattert, Germany) as previously described (Obradović et al., 2023) by adding 10^7^ CFU/mL (1 mL/bead) and incubating for 24 h at 37 °C. Beads were washed three times with phosphate-buffered saline (PBS) and either treated with individual phages or phage cocktail (phage ratio 1:1:1:1) at 10^8^ and 10^7^ PFU/mL for 3 and 24 h, respectively. Untreated beads in LB broth were used as control. Each bead was washed three times with PBS and transferred into 1 mL of PBS. Tubes were vortexed for 30 s, sonicated (Ultrasonic cleaner, USC300T, VVR International, Paris, France) for 1 min (80 W, 45 kHz) and vortexed again for 30 s to dislodge biofilm-embedded bacteria. Sonication fluids were serially diluted tenfold, and 20 μL of each dilution was plated on LB agar and incubated O/N at 37 °C for CFU counting.

### 2.9 Cesium chloride gradient purification of phage lysate

The phage lysates were purified by cesium chloride (CsCl) density gradient ultracentrifugation to remove cellular debris and endotoxins. This method was adapted from Sambrook and Russell’s Laboratory manual (Sambrook et al., 2001), with minor modifications. Briefly, 0.5 g of CsCl (Sigma-Aldrich) were dissolved in 1 mL of phage lysate, and the suspension was carefully layered onto a stepwise CsCl gradient consisting of density layers ranging from 1.20 to 1.70 g/mL in an ultracentrifuge tube (Beckman Coulter). Samples were centrifuged at 150,000 × *g* for 24 h at 4 °C in a SW41 Ti swinging-bucket rotor (Beckman Coulter). The opalescent phage band was collected using a syringe needle, transferred into a dialysis tubing with a 10 kDa MWCO (ThermoFisher), sealed with dialysis clips and supported by a floating rack, and placed for 3 h in a beaker containing 1.5 L of SM buffer, at 4 °C. The beaker was maintained under gentle agitation to allow the dialysis tubing to rotate freely, thereby facilitating CsCl dilution. The same procedure was repeated overnight in fresh SM buffer. Phage suspensions were recovered the next day, sterilized by filtration through 0.22 µm membranes and titered.

### 2.10 *In vivo* safety testing in *Galleria mellonella*

The safety of the purified phage suspensions was assessed *in vivo* using a *Galleria mellonella* model (Thiry et al., 2019a). Final-instar larvae (Italian Cricket Farm, Italy) were maintained at room temperature in the dark and starved for 24 h prior to injection (Materazzi et al., 2022). Only larvae weighing 450–600 mg and showing no signs of melanization or physical damage were selected (D’Andrea et al., 2017). The larval cuticle was disinfected with 70 % ethanol, and 10 µL of the test suspension were injected into the haemocoel *via* the last left proleg using sterile 0.3 mL syringes (BD, Franklin Lakes, NJ, USA). For each phage, both the CsCl-purified preparation and the corresponding lysate were administered at equivalent particle doses. Phages Kilian, Trimon, and Jurek were tested at 10⁸ PFU/larva, and phage Olmo at 10⁷ PFU/larva. Difference in dosage reflected differences in phage yield after purification. Control groups included larvae injected with SM buffer (10 µL/larvae) and uninjected larvae (no-treatment control). Following injection, larvae were placed in Petri dishes and incubated at 37 °C in the dark under humidified conditions without feeding. Survival was monitored daily for 6 days, with mortality defined as no response to gentle tactile stimulation and body flaccidity. Each condition comprised *n* = 5 larvae.

### 2.11 Results visualization and statistical analyses

GraphPad Prism version 10.3.1 (GraphPad Software, La Jolla, CA, USA) was used to generate graphical representations and perform statistical analyses, using Student's *t*-test and Multiple Unpaired *t*-test.

## 3 Results

### 3.1 Genome sequencing and sequence analysis of the novel *K. pneumoniae* phages

Four bacteriophages were isolated using *K. pneumoniae* ATCC BAA-2146 as the host strain. Phage Kilian, Trimon, and Olmo were isolated from river water samples, while phage Jurek was isolated from effluent water following wastewater treatment in Tuscany, Italy. Illumina™ whole-genome sequencing of phage Kilian (average coverage: 141.1 ×), Trimon (average coverage: 143.6 ×), Jurek (average coverage: 96.8 ×), and Olmo (average coverage: 27.1 ×) revealed dsDNA genomes with the highest similarity to *Klebsiella* phage cp39 (98 % query coverage and 95.19 % nucleotide identity), *Klebsiella* phage vB_Kpn-K69PH164C2 (97 % query coverage and 94.31 % nucleotide identity), *Klebsiella* phage KL (93 % query coverage and 95.50 % nucleotide identity), and *Klebsiella* phage phi1_146,002 (98 % query coverage and 97.94 % nucleotide identity), respectively. Based on these sequence homologies, phages Kilian, Trimon and Jurek belong to the *Webervirus* genus, which is classified into the taxonomic family of the *Drexlerviridae*. Those three phages constitute novel species according to the guidelines defined by the International Committee on Taxonomy of Viruses (ICTV) (Turner et al., 2021). Phage Olmo, by contrast, belongs to *Pseudotevenvirus* genus, classified into the taxonomic family of the *Straboviridae*, and is an isolate of the *Klebsiella* phage phi1_146,002 species. The genomes of phages Kilian, Trimon, and Jurek are similar in size, ranging from 49,070 to 49,774 bp, while the genome of phage Olmo is significantly larger, with a size of 176,826 bp. Intergenomic distances between phages, which confirm their genetic distinctiveness, are presented in the Supplementary Material (Fig. S1). Additional details on GC content, number of coding sequences (CDSs), tRNA genes, hypothetical proteins, and genes involved in DNA metabolism, bacterial host lysis, and structural protein functions are summarized in Table 1. A subset of coding sequences (CDSs) was also assigned under the category “other functions”. This included a putative transcriptional regulator in Kilian; a putative membrane protein and a transcriptional regulator in Trimon; one putative membrane protein in Jurek; and a broader set of proteins in Olmo. The latter comprised a co-chaperonin for GroEL, sigma factor, late promoter transcription accessory protein, LCP family protein, nucleoid disruption protein, PhoH family protein, Srd sigma factor, valyl tRNA synthetase modifier, and zeta toxin family protein. The complete genomes of the phages were deposited in GenBank under the following accession numbers: Kilian (PV340597.1), Trimon (PV340598.1), Jurek (PV360689.1), and Olmo (PV425437.1)

**Table 1:** Genome features of the novel K. pneumoniae phage genomes.

*Klebsiella* Phage	Size (bp)	GC (%)	N° tRNAs	N °CDSs	Hypothetical proteins	DNA metabolism-related proteins	Structural proteins	Host Lysis proteins	Proteins with other functions
**Kilian**	49,070	50.74	0	84	49	11	19	4	1
**Trimon**	49,685	50.69	0	84	45	13	20	4	2
**Jurek**	49,774	50.80	0	85	49	14	17	4	1
**Olmo**	176,826	44.72	2	280	161	59	43	8	9

To investigate the packing strategy used by these phages, their terminase sequences were compared with those of phages with known genome packing systems. This approach suggested a headfull packaging strategy for all four phages (Table 2).

**Table 2:** Analysis of phage packaging strategies.

Phage Name	Terminase BLASTp best hit	Query (%)	Identity (%)
**Kilian**	*Klebsiella* phage JD001 gp34	93	31.42
**Trimon**	*Klebsiella* phage JD001 gp34	93	31.23
**Jurek**	*Klebsiella* phage JD001 gp34	93	31.23
**Olmo**	*Escherichia* virus T4	97	68.91

Across the four genomes, phage Olmo differed from Kilian, Trimon, and Jurek at multiple gene modules, as suggested by its genome size being approximately three times larger (Table 1). Olmo encoded a T4-like contractile tail apparatus, including tail sheath, tail sheath stabilizer/completion proteins, tail tube protein, an expanded baseplate set (baseplate hub/distal hub, baseplate wedge and tail pin, tail tube cap, and baseplate assembly chaperone), together with fibritin and long tail fiber distal/proximal subunits and their assembly chaperones. In the other three phage genomes, the genes encoding the sheath and baseplate components are absent, while tape-measure proteins and multiple minor tail proteins are encoded. These findings suggested a myoviral morphotype with a contractile tail for Olmo, while the other phages display a siphoviral morphology with non-contractile tails. All four genomes encoded terminase small/large subunits, a portal protein, major capsid and head morphogenesis factors; in phage Olmo, these were enriched by head completion, prohead core/scaffolding, head assembly chaperone, and capsid vertex/outer-capsid proteins. Replication and nucleotide metabolism module further distinguished Olmo from the other phages (58 and 11–14 CDSs, respectively, as show in Table 1). Indeed, this phage carried multiple DNA helicases, DNA ligase, RNA ligases, dUTPase, 5′–3′ deoxyribonucleotidase, polynucleotide kinase, NudE/Nudix hydrolase, phosphatase, DNA polymerase, topoisomerase II, primase subunit, single-stranded DNA-binding protein (SSB), ribonucleotide reductase class I α/β and class III (activating protein and large subunit), and the full dCMP→dTMP set. By contrast, Kilian, Trimon, and Jurek retained an essential repertoire (helicases, primase, SSB, exonuclease, polynucleotide kinase; dNMP kinase in Kilian and Jurek), and lacked ribonucleotide reductase (RNR) and the explicit dTMP pathway, with Trimon additionally encoding a second DNA helicase, a nucleoside triphosphate hydrolase, and a viral replication and repair endonuclease domain. Only Olmo encoded a defined late-transcription takeover set, including a predicted sigma factor for late transcription, late-promoter accessory proteins, regA (translational repressor), RNA-polymerase-binding and nucleoid disruption proteins, whereas the other phage genomes contain at most a transcriptional regulator. Concerning DNA modification and, in particular, the presence of DNA methyltransferases, Dam was present in all phages, Dcm was present in Kilian and Trimon (Jurek encodes a generic DNA methyltransferase), and Olmo included additional DNA methyltransferases; recombination and genome plasticity markers were also enriched in Olmo, which encoded UV-sensitive gene Y (a T4-like recombination mediator protein), multiple homing endonucleases (including HNH/Seg-like) and endonuclease VII. Finally, Olmo carried two tRNAs (tRNA-Gly-TCC, tRNA-Met-CAT), absent from the other three genomes (Table 1). All four phages encoded the holin–endolysin–spanin triad for lysis of Gram-negative hosts (Cahill and Young, 2019), but a lysis-inhibition markers (rI/rII-like) only present in Olmo.

Importantly, genome annotation revealed no genes associated with lysogeny, such as integrases or repressors, suggesting that all phages exhibit a lytic lifecycle. Furthermore, no genes encoding proteins associated with bacterial antibiotic resistance or virulence factors were identified.

**Fig. 1:**
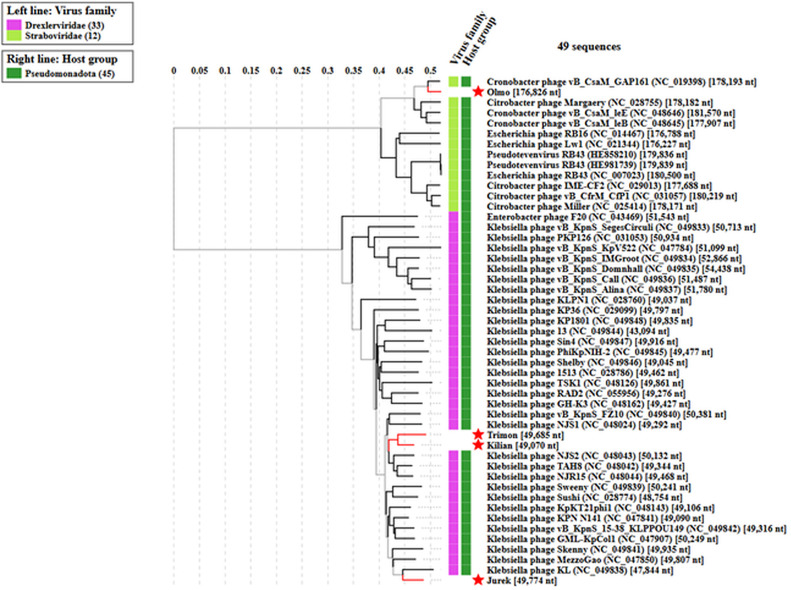
Phylogenetic tree of Kilian, Trimon, Jurek and Olmo phages generated by VipTree.

The phylogenetic analysis conducted using the VipTree bioinformatics tool revealed that phage Kilian, Trimon, and Jurek cluster within the same clade, whereas Olmo belongs to a different clade (Fig. 1).

Collectively, the genomic and functional features clearly distinguish phage Olmo from Kilian, Trimon, and Jurek, supporting its classification as a member of a different family and reflecting two distinct evolutionary lineages within *Klebsiella*-infecting phages.

### 3.2 Morphological characterization of *K. pneumoniae* phages by transmission electron microscopy (TEM)

**Fig. 2:**
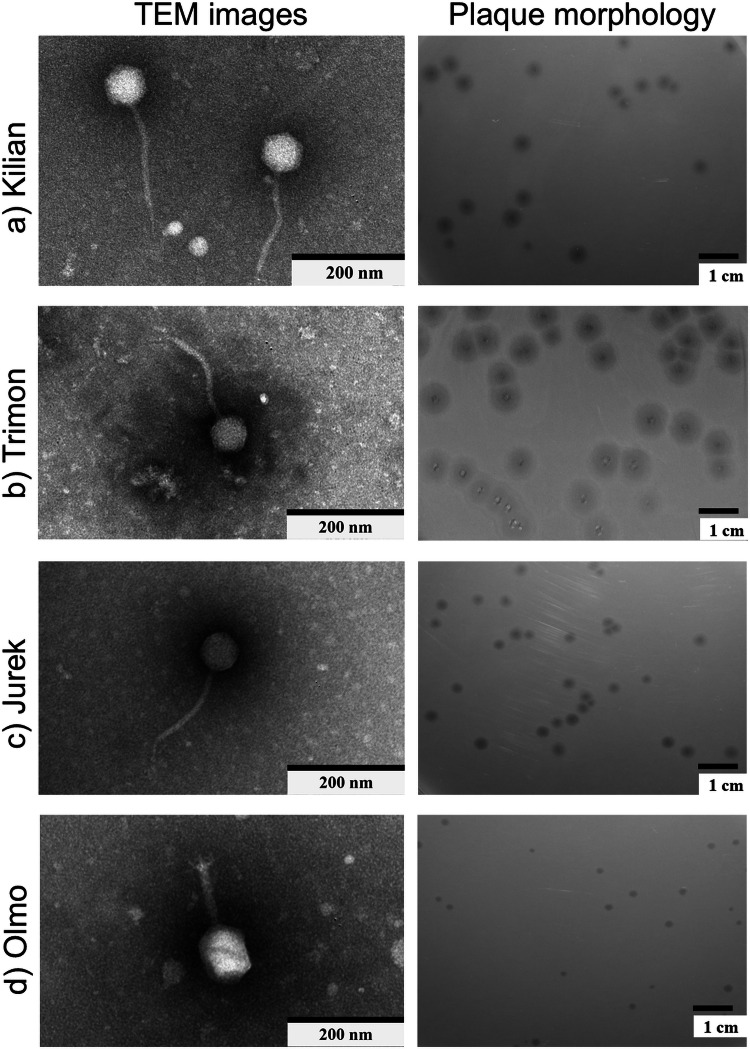
Transmission electron microscopy imaging and corresponding plaque morphology of phage (a) Kilian, (b) Trimon, (c) Jurek and (d) Olmo. The scale bar corresponds 200 nm and to 1 cm for plaque morphology images obtained by plaque assay.

TEM analysis confirmed that all phages belong to the *Caudoviricetes* class (Fig. 2). Phages Kilian, Jurek, and Trimon displayed a siphoviral morphology characterized by long, non-contractile tails, with lengths of approximately 202 nm, 175 nm, 160 nm, respectively. Phage Olmo exhibited a myoviral morphotype with a contractile tail (length ≈ 124 nm). All phages showed icosahedral capsids, with diameters of approximately 101 nm, 65 nm, 63 nm, 63 nm, respectively. The baseplate diameter was also evaluated for Kilian ≈ 8 nm, Trimon ≈ 7 nm, Jurek ≈ 6 nm and Olmo ≈ 35 nm.

The plaques morphologies were comparable in appearance for phages Kilian (1.5 mm), Trimon (6.6 mm) and Jurek (2.7 mm), although they differed in size, with prominent halos observed for Kilian (1 mm), Trimon (2.7 mm), and Jurek (0.8 mm). Phage Olmo produced smaller plaques compared to the other phages (0.4 mm diameter).

### 3.3 Phage adsorption and one-step growth curve analysis

**Fig. 3:**
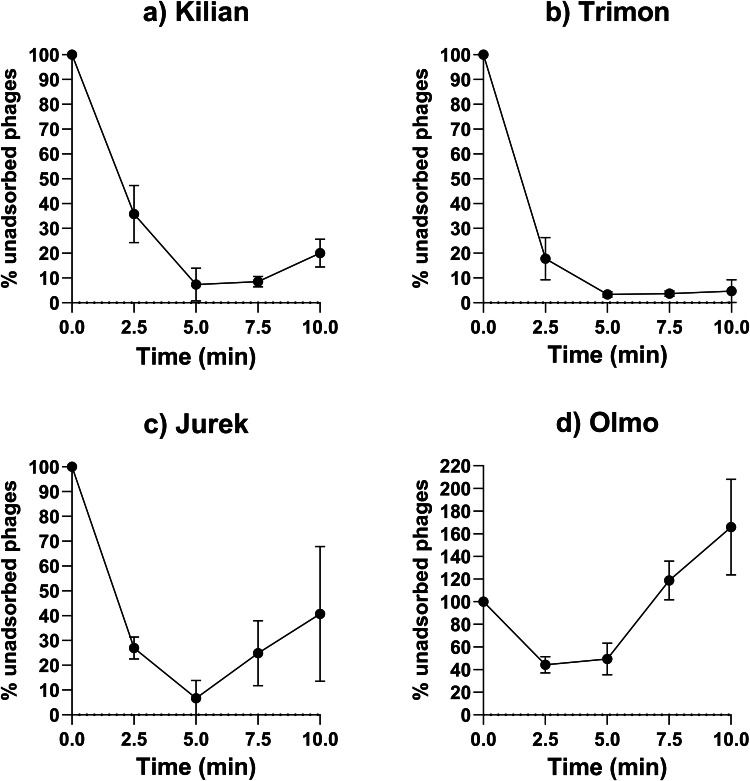
Adsorption assay of phage (a) Kilian, (b) Trimon, (c) Jurek and (d) Olmo. Data represent three independent experiments, each performed in duplicate.

To investigate both the interaction and the replication cycle of the four phages against their bacterial host (*K. pneumoniae* ATCC BAA-2146), phage adsorption assays and one-step growth curves were performed. As shown in Fig. 3, results are expressed as the percentage of unabsorbed phages over time. Phage Kilian, Trimon, and Jurek exhibited maximum adsorption at five minutes post-infection, with 92.67 %, 96.67 %, and 93.31 % of adsorbed viral particles, respectively. In contrast, phage Olmo showed a maximum viral particle adsorption of 55.75 % at 2.5 min.

**Fig. 4:**
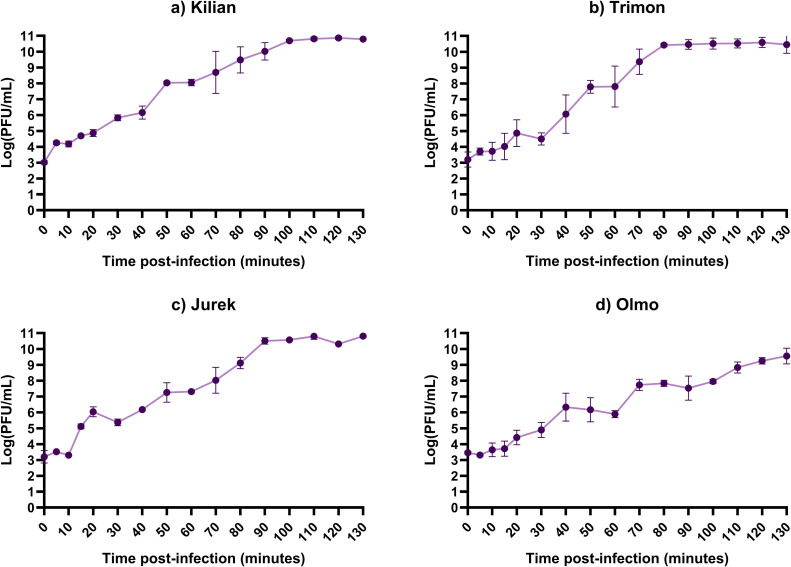
One-step growth curves of phage (a) Kilian, (b) Trimon, (c) Jurek, and (d) Olmo. Data represent three independent experiments, each performed in duplicate.

The one-step growth curve indicated a latent period for phage Trimon and Olmo of approximately 15 min, with a burst size of about 69 and 22 PFUs/CFU, respectively (Fig. 4). Conversely, the latent periods of Kilian and Jurek were shorter (5 and 10 min, respectively). These two phages also exhibited higher burst sizes, with Kilian producing approximately 474 virions per infected bacterial cell and Jurek producing around 196 PFUs/CFU. The complete replication cycle for phage Olmo was approximately 130 min, for phage Kilian, Trimon and Jurek were 100, 80 and 90 min, respectively.

### 3.4 Determination of host range and efficiency of plating (EOP) of *K. pneumoniae* phages

**Fig. 5:**
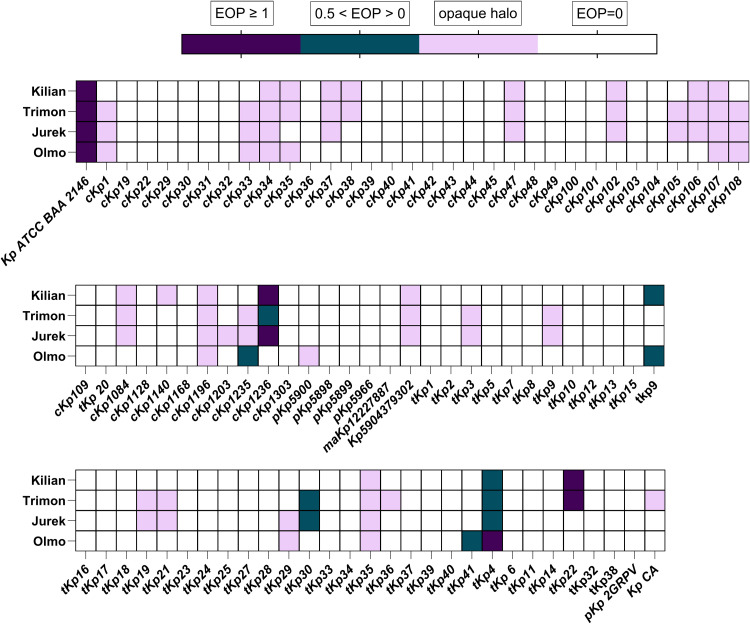
Efficacy of plating of phage Kilian, Trimon, Jurek and Olmo against 90 K. pneumoniae strains. Data represent two independent experiments, each performed in duplicate.

To evaluate the ability of phages to infect and replicate in *K. pneumoniae* strains, EOP and the amplification in liquid culture of selected phages were carried out. The four phages exhibited a narrow host range when tested against a panel of 89 clinical strains of K. pneumoniae and the host Kp ATCC BAA-2146 (Fig. 5). Phage Kilian was active against four strains: cKp1236 (ST37, KPC), tKp9 (KPC), tKp4 (VIM) and tKp22 (VIM). Phage Trimon displayed activity against four strains: cKp1236, tKp4, tKp30 and tKp22. Phage Jurek was effective against three strains: cKp1236, tKp30 and tKp4. Lastly, phage Olmo showed activity against four strains: cKp1235 (ST512, KPC), tKp9, tKp41(KPC) and tKp4.

For 27 strains of *K. pneumoniae*, a diffused opaque halo of lysis was observed, without the presence of any single plaques at lower titers. This phenomenon might be due a lysis from without occurring in the absence of productive phage infection (Abedon, 2011). To determine whether phage infection was occurring in these strains, liquid infection assays at an MOI of 0.1 were performed in LB broth, and phage titers were evaluated after 24 h to assess any increase. Upon titration of the bacteriophages, it was observed that for six of the tested strains (Table 3), the number of plaques formed by at least one of the phages under study exceeded the initial inoculum titer, indicating that a phage propagation within those bacterial strains occurred.

**Table 3:** Log₁₀ increase in phage titers at 24 h post-infection against K. pneumoniae clinical isolates. Data represent two independent experiments, each performed in duplicate.

K. pneumoniae strain	Kilian	Trimon	Jurek	Olmo
cKp1	/	/	2.81	0.98
cKp37	2.04	/	/	/
cKp43	2.08	/	/	/
cKp106	1.65	0.79	/	/
tKp19	/	1.38	/	/
tKp22	/	/	3.52	/

To probe cross-genus host specificity, phages were tested against a panel of 14 *E. coli* strains by spot assay. No lytic zones were observed for any phage–strain combination, indicating no detectable activity on *E. coli*.

### 3.5 *In vitro* lytic activity of *K. pneumoniae* phages and their combination in a phage cocktail

**Fig. 6:**
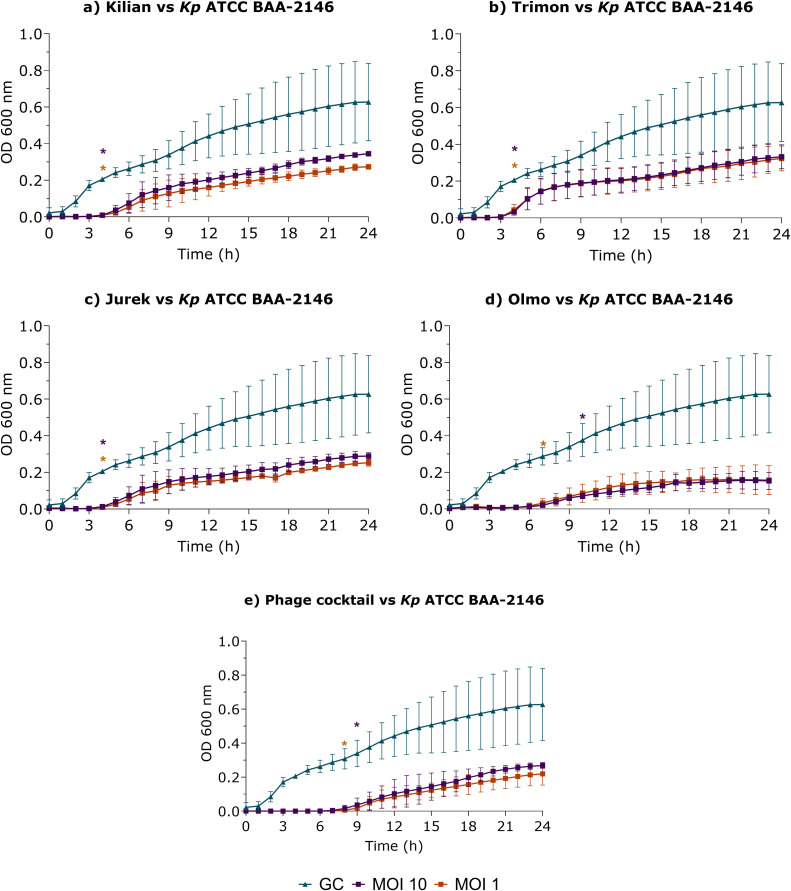
Optical density at 600 nm (OD_600_) measurements of K. pneumoniae ATCC BAA-2146 treated with phage (a) Kilian, (b) Trimon, (c) Jurek (d) Olmo and (e) their combination at MOI 1 (orange curves) and 10 (purple curves). Blue curves indicated bacterial growth control (GC). The OD_600_ was measured hourly over a 24-h period. Data represent three independent experiments, each performed in quadruplicate. A multiple unpaired *t*-test was used to assess significance (*, p-value < 0.05), with significant differences indicated in orange (MOI 1) and purple (MOI 10).

**Fig. 7:**
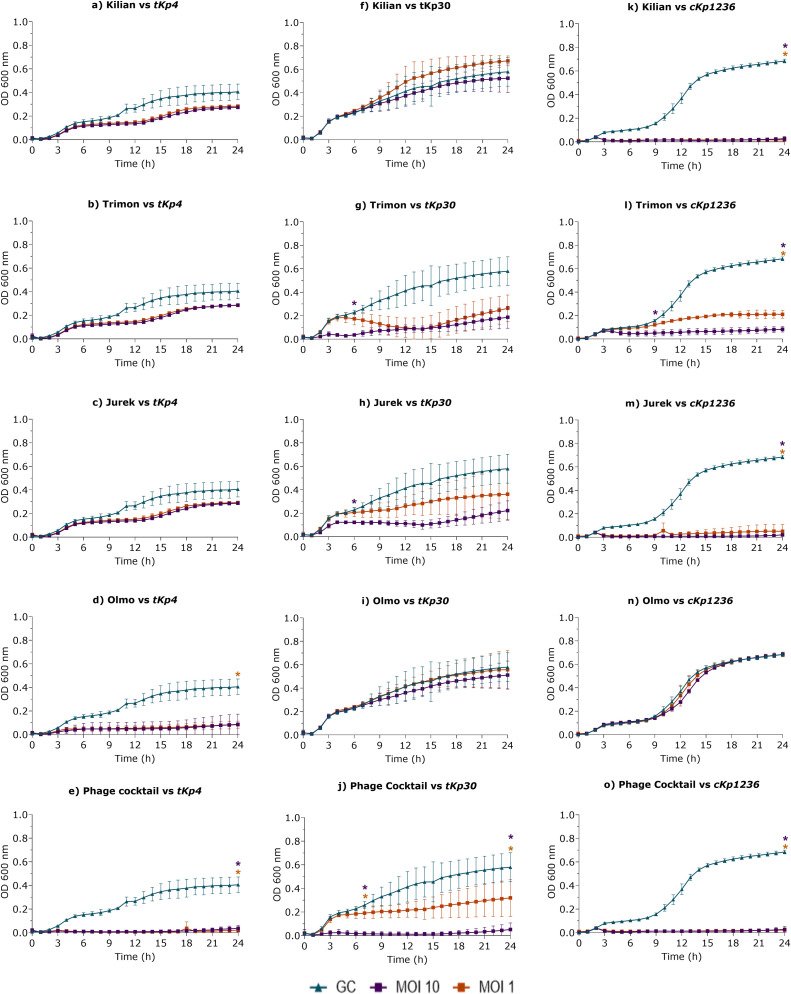
Optical density at 600 nm (OD_600_) measurements of K. pneumoniae tKp4, tKp30, cKp1236 clinical isolates treated with phage a,f,k) Kilian, b,g,l) Trimon, c,h,m) Jurek d,i,n) Olmo and e,j,o) the four-phage cocktail at MOI 1 (orange curves) and 10 (purple curves). Blue curves indicate bacterial growth control (GC). OD_600_ was measured hourly over a 24-h period. Data represent three independent experiments, each performed in quadruplicate. A multiple unpaired *t*-test was used to assess significance (*, p-value < 0.05), with significant differences indicated in orange (MOI 1) and purple (MOI 10).

The ability of the four phages tested individually and in combination, to infect and lyse *K. pneumoniae* ATCC BAA-2146 (Fig. 6) and clinical isolates (Fig. 7) was evaluated by measuring OD_600_ every hour over a 24-h period.

As shown in Fig. 6, the individual treatments of *K. pneumoniae* ATCC BAA-2146 with phage Kilian, Trimon, and Jurek resulted in statistically significant lower OD_600_ values up to four hours, at both MOI tested, compared to the untreated control. In contrast, the difference in OD_600_ measurements between bacteria treated with phage Olmo in comparison to the untreated control was statistically significant up to seven hours at MOI 1 and up to 10 h at MOI 10. Subsequently, OD_600_ values increased, reaching levels comparable to the untreated bacterial growth control. Furthermore, to assess an increase in antimicrobial efficacy, the four phages were combined in equal proportions to prepare a phage cocktail, which was tested against the bacterial host. Under this condition, OD_600_ measurements remained low, at the baseline, with a statistically significant difference in terms of OD_600_ between treated bacteria and untreated control up to eight hours at MOI 1 and up to nine hours at MOI 10.

Following the evaluation of phage cocktail activity against the host strain, the lytic kinetics of phages Kilian, Trimon, Jurek, and Olmo were also assessed, both individually and as a cocktail, against clinical isolates susceptible to at least two phages by EOP test. Fig. 7 showed lysis curves for the three clinical isolates in which the phage cocktail led to an OD_600_ reduction compared to the growth control.

For the clinical isolate *K. pneumoniae tKp4* (Fig. 7a-e), OD₆₀₀ measurement after treatment with phage Kilian, Trimon and Jurek at both tested MOIs, as well as Olmo at MOI 10, showed values comparable to those of the untreated control. This suggests that, individually, these phages were not effective in controlling bacterial growth.

Conversely, treatment with phage Olmo at MOI 1 and the phage cocktail at both MOIs resulted in a statistically significant reduction in OD₆₀₀ values compared to untreated controls throughout the 24-h period, indicating effective inhibition of bacterial growth under these experimental conditions.

Regarding the *K. pneumoniae tKp30* strain (Fig. 7F-j), OD₆₀₀ measurements after treatments with phage Kilian, Olmo at both MOIs, and Trimon and Jurek at MOI 1 showed values comparable to those of the bacterial growth control, demonstrating limited individual efficacy against this isolate. However, treatment with phages Trimon and Jurek at MOI 10 resulted in a statistically significant OD_600_ reduction between 4 and 6 h, suggesting earlier timing efficacy under these conditions. Notably, the combination of all four phages (Fig. 7j) led to a significant reduction in OD₆₀₀ values compared to the growth control between 4 and 7 h and again between 20 and 24 h. This result demonstrates a strong antimicrobial effect at both MOIs and highlights the enhanced efficacy of the phage cocktail over individual treatments for *K. pneumoniae tKp30* strain.

*In vitro* lytic activity of phages Kilian, Trimon, and Jurek, tested individually at MOI 1 and 10 against the *K. pneumoniae cKp1236* strain (Fig. 7k-o), demonstrated a significant efficacy when used alone, as indicated by a statistically significant reduction in OD₆₀₀ values between 3 and 24 h. In contrast, phage Olmo showed no effectiveness over time, as OD₆₀₀ measurements over the 24-h period remained comparable to those of the untreated growth control. The combination of all four phages resulted in a statistically significant reduction in OD₆₀₀ values compared to the untreated growth control between 3 and 24 h.

### 3.6 Antibiofilm efficacy of *K. pneumoniae* phages and phage cocktail

**Fig. 8:**
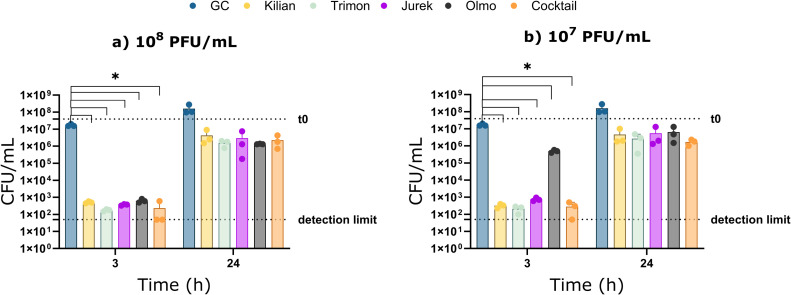
Antibiofilm activity of the four individual phages (Kilian, yellow bars; Trimon, light green bars; Jurek, purple bars; and Olmo, grey bars) and the phage cocktail (orange bars) against K. pneumoniae ATCC BAA-2146, evaluated by measuring the CFU/mL at 3 and 24 h post incubation. Bacterial biofilms were treated with either 10^8^ PFU/mL (a) or 10^7^ PFU/mL (b) of each phage or the cocktail. GC (blue bars) represents the growth control. T0 dashed line represent CFU/mL after biofilm formation. Data represent three independent experiments, each performed in duplicate. A multiple unpaired *t*-test was used to assess significance; *, p-value <0.05.

The antibiofilm activity of individual phages and the cocktail was evaluated against biofilm-embedded K. pneumoniae, preformed on porous glass beads. As shown in Fig. 8, a strong reduction in CFU/mL (> 4-log_10_ units) was observed in biofilms treated with phages, both individually and in combination, at 3 h post-treatment, in comparison to the untreated control. The effect was independent of the titer tested, except for Olmo treatment at 10^7^ PFU/mL, where a reduction of 2-log_10_ was observed. In contrast, after 24 h, both single-phage and cocktail treatments resulted in about 1.5-log_10_ reduction in CFU/mL at both phage concentrations in comparison to the untreated control.

### 3.7 *In vivo* safety assessment of phages purified through cesium chloride (CsCl) gradient purification

**Fig. 9:**
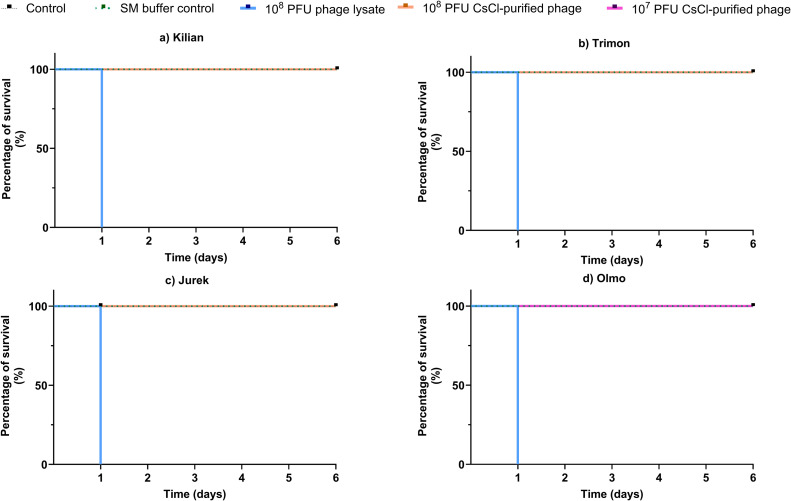
Survival of Galleria mellonella after injection of phage (a) Kilian, (b) Trimon, (c) Jurek, and (c) Olmo, respectively. Curves report percentage survival over 6 days. Controls: uninjected control (black, dashed line) and SM buffer (green, dotted line). Treatments: 10^8^ PFU phage lysate (blue), 10^8^ PFU CsCl-purified phage (orange), and 10^7^ PFU CsCl-purified phage (pink).

To evaluate the safety of the phages, they were purified using a CsCl gradient and subsequently tested in *Galleria mellonella* larvae. After CsCl gradient purification, maximum recoverable titers reached 10^10^ PFU/mL for phages Kilian, Trimon, and Jurek, and 10^9^ PFU/mL for Olmo. Consequently, the first three phages were tested at 10^8^ PFU/larva, while Olmo was injected at 10^7^ PFU/larva. Injections of non-purified lysates of each phage at all tested concentrations resulted in 100 % larval mortality within 24 h, as shown in Fig. 9. In contrast, all larvae injected with CsCl-purified preparations survived throughout the entire 6-day observation period, showing outcomes identical to those of larvae injected with SM buffer or left untreated.

## 4 Discussion

Bacteriophages are increasingly regarded as a promising alternative or adjunct therapeutic option, particularly in cases where antibiotics fail to eradicate bacterial infections. This growing interest is reflected in the expanding body of research assessing the efficacy and safety of phage applications in animal studies, case reports, and clinical trials (Kulshrestha et al., 2024). The use of bacteriophages in clinical settings, known as bacteriophage therapy, has shown significant promise in fighting MDR bacterial infections, including those caused by *K. pneumoniae* (Le Bris et al., 2025; Zaki et al., 2023)*.*

In this study, four bacteriophages (Kilian, Trimon, Jurek, and Olmo) isolated from Tuscany rivers and wastewaters were isolated and characterised, using the NDM-producing strain *K. pneumoniae* ATCC BAA-2146 as host. All the characterized phages exhibited a strictly lytic nature, an essential feature for therapeutic applications. Phylogenetic and genome analyses, along with TEM imaging, revealed that three out of four phages (Kilian, Trimon, and Jurek) are siphoviruses, identified as new species, and are closely related, while Olmo is a myovirus belonging to a different clade. Notably, no phages showed sequences in their genomes that could be linked to antibiotic resistance, virulence, or toxicity genes. Interestingly, when analysing plaque morphology, phage Kilian, Trimon and Jurek produced clear plaques surrounded by distinct and diffuse halos, suggesting the possible production of depolymerases, enzymes that degrade bacterial surface polymers, aiding phage infection (Domingo-Calap et al., 2020). Capsular depolymerases might also be used as key components in new biotechnological therapies designed to degrade biofilms, as demonstrated in previous studies (Asif et al., 2023; Domingo-Calap et al., 2020).

In the context of phage therapy, phages with rapid adsorption, short latent period and high burst size are optimal candidates for clinical use (Forti et al., 2018). Overall, the phages characterised in this study exhibited adsorption times of 2.5–5 min, latency periods of 5–15 min and burst sizes ranging from 22 to 474 PFU/cell. Mechanistically, Olmo’s genomic distinctiveness is mirrored by phenotypic readouts across key infection steps. For instance, Olmo adsorbed less efficiently than the other three phages. Indeed, in T4-like phages, adsorption typically proceeds *via* reversible binding of the long tail fibers followed by an irreversible baseplate rearrangement that triggers tail-sheath contraction and DNA delivery; when capsular processing at the fiber tips is ineffective, fewer particles progress to this irreversible step, yielding lower net adsorption (Hu et al., 2015; Kanamaru et al., 2002; Leiman and Shneider, 2012). Moreover, its larger genome and expanded tail/baseplate and head toolkits could imply higher assembly costs, consistent with the longer latent period and smaller burst size observed relative to the other phages (Rao et al., 2023). The life cycles parameters are comparable or higher than those reported for several *Klebsiella* infecting phages in the literature, such as vB_KpnS_SXFY507 (246 PFU/cell) (Feng et al., 2023), φBO1E (300 PFU/cell) (D’Andrea et al., 2017), SJM3 and LASTA (155 and 187 PFU/cell, respectively, with adsorption times of 5 min each) (Leiman and Shneider, 2012) and GP-4 and GP-5 (53 and 87 PFU/cell, respectively) (Ponsecchi et al., 2024). Furthermore, the short latency periods observed for the phages characterised in this study contrast favourably with the longer phases reported for phages such as GP-1 (50 min) (Ponsecchi et al., 2024), SJM3 and LASTA (80 min each) (Obradović et al., 2023) and were in line with GP-2, GP-4, GP-5 (10–15 min) (Ponsecchi et al., 2024) and φBO1E (10 min) (D’Andrea et al., 2017).

The phages displayed a narrow spectrum of activity across the 90 K*. pneumoniae* tested strains. Specifically, Kilian was active against 8.9 % of the strains, Trimon against 7.8 %, Jurek against 6.7 %, and Olmo against 6.7 %. Overall, 14.4 % of the bacterial strains tested were susceptible to at least one phage, including strains belonging to ST11, ST512, ST147, ST37, and ST307 clones, the most epidemiologically relevant MDR *K. pneumoniae* strains, regardless of the production of specific carbapenemases. These values reflect not only results from EOP assays on solid media, but also data on phage replication in liquid culture. Acknowledging the limitations of this approach and performing liquid infection assays for strains with ambiguous results were essential for identifying six additional *K. pneumoniae* strains susceptible to at least one phage. On the other hand, a substantial proportion of the *K. pneumoniae* strains were not susceptible to the phages characterized in this study. The lack of susceptibility may be due either to the absence of specific receptors required for phage adsorption or to the presence of bacterial defence mechanisms, such as CRISPR–Cas systems (Li et al., 2018; Shen et al., 2017), restriction–modification (Lee et al., 1997), and abortive infection (Abi) mechanisms, including cyclic oligonucleotide-based anti-phage signalling systems (CBASS) (Ortiz-Cartagena et al., 2025). This could also be supported by the observation of faint lysis zones without productive phage propagation associated with clear plaques forming units (Watson et al., 2019). We also tested the four *Klebsiella* phages against a panel of 14 *E. coli* clinical isolates, another Gram-negative bacterium belonging to *Enterobacteriales* (Janda and Abbott, 2021). As expected, no lytic activity was observed against any strain, confirming the host specificity of the phages. However, the lack of activity against a small panel of *E. coli* isolates does not, by itself, exclude the susceptibility of this bacterium to phages or cross-genus infectivity within *Enterobacteriales* (*e.g., Enterobacter, Citrobacter, Salmonella, Shigella, Cronobacter*) (Adeolu et al., 2016). Systematic testing across additional genera within this family would therefore be required to substantiate a species- or genus-restricted profile.

In this study, we combined the four phages into a cocktail to assess their overall activity against selected clinical *K. pneumoniae* isolates, following a precision phage therapy approach in which phages are chosen according to the patient’s strain. For further investigation, we consider crucial to study the phage receptors, as well as the capsular types recognized by each phage, enabling their use as modular components of rationally designed cocktails, avoiding redundancy, with the aim of preparing broad-spectrum phage cocktails (Abedon et al., 2021; Ferriol-González et al., 2024; Wyres et al., 2020). These can be integrated with the use of additional phages or capsular depolymerases, not only to broaden host range, but also to improve receptor access and limit the emergence of phage resistance (Zurabov et al., 2023).

Guided by this premise, in phage therapy it is also important to monitor the lytic activity of phages over time to assess their therapeutic potential and to detect any antagonistic interactions. In this study, we present the killing kinetics of each individual phage, as well as the four-phage cocktail, against the host strain *K. pneumoniae* ATCC BAA-2146 and three clinical isolates (*tKp4, tKp30*, and *cKp1236*). Comparison of the *in vitro* lytic activity of individual phages against clinical isolates and the corresponding EOP data revealed different levels of concordance. In the case of *cKp1236* and *tKp4*, there was a clear concordance: phages with high EOP values also showed strong and prolonged lytic effects in liquid culture, whereas phages with low EOP performance corresponded to control of bacterial load over time. In contrast, the correlation was less linear for *tKp30* strains. A similar pattern was observed for *tKp30*: although the EOP values for most phages were low or undetectable, Trimon and Jurek at high MOI exerted a transient inhibitory effect in the early phase of incubation, while the phage cocktail showed a distinct lytic profile with significant reductions in OD_600_ in both the early and late phases. These results suggest that EOP assays alone may underestimate the therapeutic potential of some phages, particularly in liquid or dynamic environments that more closely mimic *in vivo* conditions. They also highlight the added value of phage combinations, which can exploit partial or undetectable individual activities and convert them into robust and clinically relevant antibacterial effects through potential synergistic interactions. Although all phages were isolated using the same bacterial host and three of them were genomically close, they displayed slightly distinct host ranges and lytic profiles. This supports the rationale for their combined use, suggesting that, while they may target the same bacterial surface receptor, they could interact with different portions of that receptor. Such differential interactions may underlie the synergistic effect observed *in vitro* against three clinical *K. pneumoniae* strains. A similar approach was described by Forti and colleagues, where closely related phages with <8 % genomic divergence and exhibiting differing host ranges were exploited for phage combination, supporting the strategy to enhance therapeutic efficacy (Forti et al., 2018). Furthermore, the use of a cocktail in clinical settings may overcome the emergence of phage resistance (Hatfull et al., 2022). Indeed, the phage cocktail used in this study maintained low OD₆₀₀ values over 24 h. Conversely, single phages showed an increase of OD₆₀₀ values, suggesting the emergence of phage-resistant mutants.

This study also explored the antibiofilm activity of the phages, both individually and in combination as a cocktail. At three hours post-treatment, a strong reduction in CFU/mL was observed for all phages compared to the untreated control, when tested either individually or in combination. More specifically, of the four phages, Olmo showed the weakest antibiofilm activity. This may reflect its halo-free plaque morphology, in contrast to the three siphoviruses, which produce halos and performed better in biofilm assays. However, the regrowth of sessile bacteria after 24 h of phage exposure suggested the emergence of phage-resistant mutants, as previously reported in studies aiming at preventing the development of phage resistance in *K. pneumoniae* biofilms (Chen et al., 2024b; Zurabov et al., 2023). As shown in various studies, the use of phages in combination with antibiotics may offer a promising therapeutic approach for treating biofilm-related infection (Cesta et al., 2023; Rubalskii et al., 2020). These findings are particularly relevant for the management of prosthesis-related infections and infections associated with prolonged catheter use, where biofilm formation poses a major clinical challenge.

Data from animal experiments are essential to assess safety of phage therapy, and *G. mellonella* provides a first and pragmatic bridge between *in vitro* assays and mammalian models (Serrano et al., 2023; Tsai et al., 2016). In our study, *G. mellonella* provided an early toxicovigilance readout, highlighting that, to advance toward *in vivo* efficacy testing, phage preparations should be adequately purified to minimize endotoxin and other bacterial contaminants (Hietala et al., 2019). To this end, we obtained high-titer phage stocks though CsCl gradient purification, which were presumably less contaminated, as indicated by the longer larval survival compared to the lysate. In previous *G. mellonella* studies, phage preparations have been processed with two purification method, PEG precipitation followed by CsCl gradient ultracentrifugation (Thiry et al., 2019a) or only by CsCl gradient ultracentrifugation (Han et al., 2023), and larvae survived phage administration in both cases. In contrast, the *K. pneumoniae* phage ϕBO1E was administered as a filtered lysate in SM buffer, without further purification. However, the phage lysate did not affect larval survival (D’Andrea et al., 2017). The absence of mortality despite the use of an unpurified lysate may be due to the limited amount of bacterial contaminants in the preparation, likely reflecting the low endotoxin content or reduced toxicity of the *K. pneumoniae* strain used for phage propagation. In our case, since the phage lysate led to larval death within 24 h, further purification was required. CsCl gradient ultracentrifugation was selected for its high efficiency in endotoxin removal (Van Belleghem et al., 2017). To conclude, while *G. mellonella* is a sensitive and valid first indicator, it does not recapitulate mammalian pharmacokinetics (PK) and pharmacodynamics (PD) or adaptive immune responses (Ménard et al., 2021). Therefore, the safety data should trigger confirmatory studies in mouse infection models using preparations that meet quantitative release criteria (*e.g.*, acceptable endotoxin levels in EU/mL and minimal residual host DNA/protein carryover) (Penziner et al., 2021).

In conclusion, our comprehensive genotypic and phenotypic characterisation of the presented bacteriophages, combined with their promising *in vitro* activity against carbapenem-resistant *K. pneumoniae* strains, provides a solid foundation for their potential therapeutic application in clinical settings. However, further assays *in vivo* models may be useful to determine PK and PD parameters in a more complex biological environment.

## Funding sources

This research was funded by (i) PNRR THE e Tuscany Health Ecosystem; Spoke 7—Innovating Translational Medicine-Sub-project 5—Innovative models for management of infections caused by antibiotic-resistant bacteria (Project code: ECS00000017; CUP I53C22000780001); (ii) PNRR; SPOKE 5- Inflamatory and infectious disease—Sub-project 4; WP 2-Innovative strategies to couteract novel antimicrobial resistant bacteria and viral infection From Research to Business” – Investment 1.4: Strengthening research infrastructures and creating national R&D leaders in selected Key Enabling Technologies. (MUR CN_00000041); (iii) Hub multidisciplinare e interregionale di ricerca e sperimentazione clinica per il contrasto alle pandemie e all’antibioticoresistenza (PAN—HUB)” funded by the Italian Ministry of Health (POS 2014–2020, project ID: T4-AN-07, CUP: I53C22001300001)

## CRediT authorship contribution statement

**Elisa Fausti:** Conceptualization, Data curation, Formal analysis, Investigation, Methodology, Visualization, Writing – original draft, Writing – review & editing. **Andrea Bonacorsi:** Formal analysis, Methodology, Writing – original draft, Writing – review & editing. **Novella Cesta:** Conceptualization, Formal analysis, Validation, Writing – review & editing. **Cesira Giordano:** Formal analysis, Methodology, Writing – review & editing. **Simona Barnini:** Formal analysis, Methodology, Writing – review & editing. **Magda Marchetti:** Formal analysis, Methodology, Writing – original draft, Writing – review & editing. **Claudia Campobasso:** Formal analysis, Methodology, Writing – review & editing. **Rob Lavigne:** Validation, Writing – review & editing. **Anna Altieri:** Methodology, Writing – review & editing. **Cartesio D’Agostini:** Methodology, Writing – review & editing. **Marco Iannetta:** Supervision, Validation, Writing – review & editing. **Vincenzo Malagnino:** Methodology, Writing – review & editing. **Arianna Tavanti:** Funding acquisition, Supervision, Writing – review & editing. **Loredana Sarmati:** Funding acquisition, Supervision, Writing – review & editing. **Mariagrazia Di Luca:** Conceptualization, Formal analysis, Funding acquisition, Project administration, Supervision, Writing – original draft, Writing – review & editing.

## Declaration of competing interest

The authors declare the following financial interests/personal relationships which may be considered as potential competing interests:

Mariagrazia Di Luca reports financial support was provided by Italian Ministry of University and Research (MUR). Loredana Sarmati reports financial support was provided by Italian Ministry of University and Research (MUR). Arianna Tavanti reports financial support was provided by Italian Ministry of Health. If there are other authors, they declare that they have no known competing financial interests or personal relationships that could have appeared to influence the work reported in this paper.

## Appendix Supplementary materials

Image, application 1
