# Supplementary material for: Characterisation of four novel bacteriophages targeting carbapenem-resistant Klebsiella pneumoniae and their lytic activity alone and in combination
Source: Curr Res Microb Sci. 2025 Nov 10;9:100509. doi: 10.1016/j.crmicr.2025.100509 (PMC12666361; doi:10.1016/j.crmicr.2025.100509)
Supplement: Supplementary file 1 [file mmc1.docx]

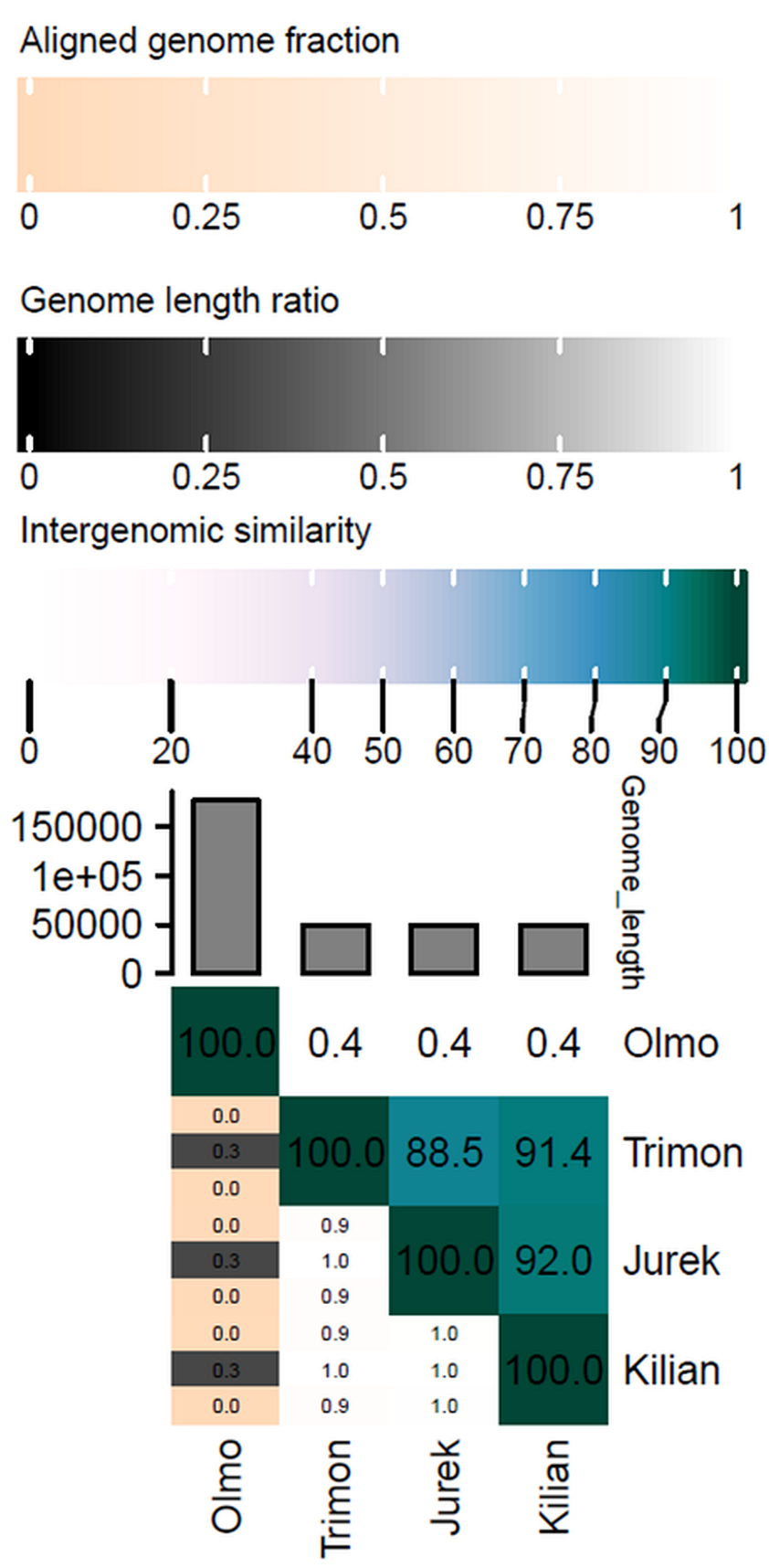
**Supplementary data**

**Figure S1.** *Intergenomic similarity between phage Kilian, Trimon, Jurek and Olmo.*
